# Supplementary figures and images for: Rescuable sleep and synaptogenesis phenotypes in a Drosophila model of O-GlcNAc transferase intellectual disability
Source: eLife. 2024 Nov 13;13:e90376. doi: 10.7554/eLife.90376 (PMC11623933; doi:10.7554/eLife.90376)

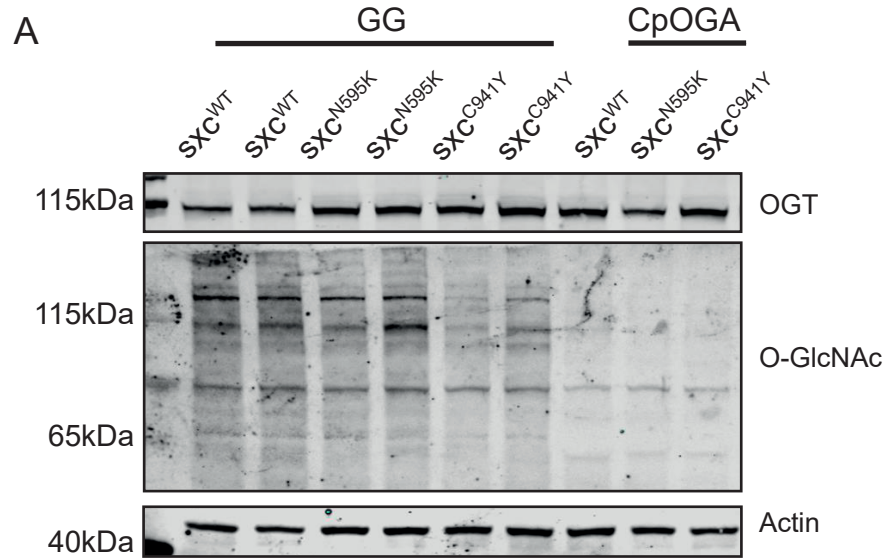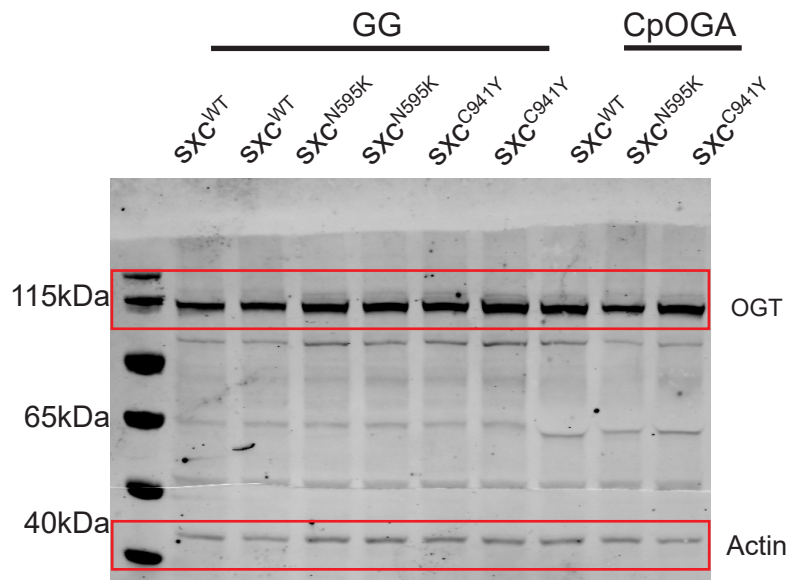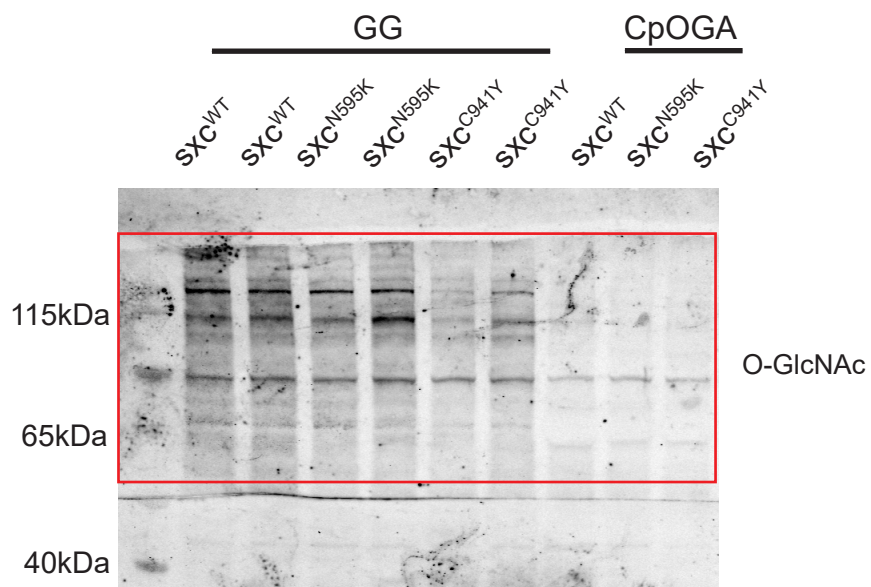

Supplement: Figure 1—source data 3. [file elife-90376-fig1-data3.zip › 1A_uncropped.pdf]

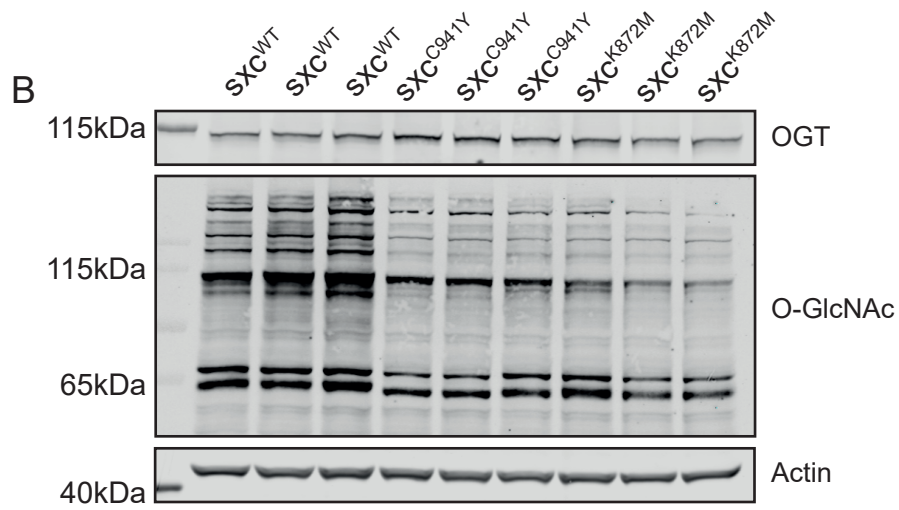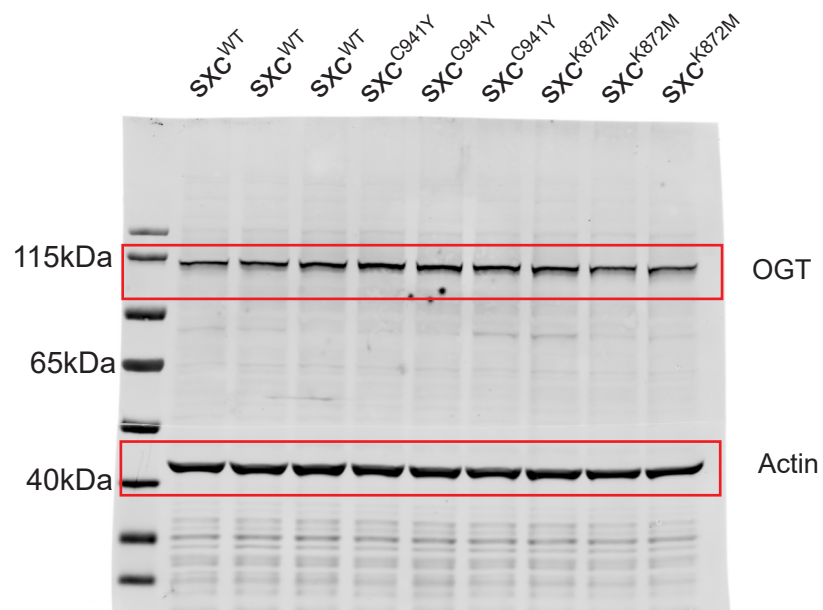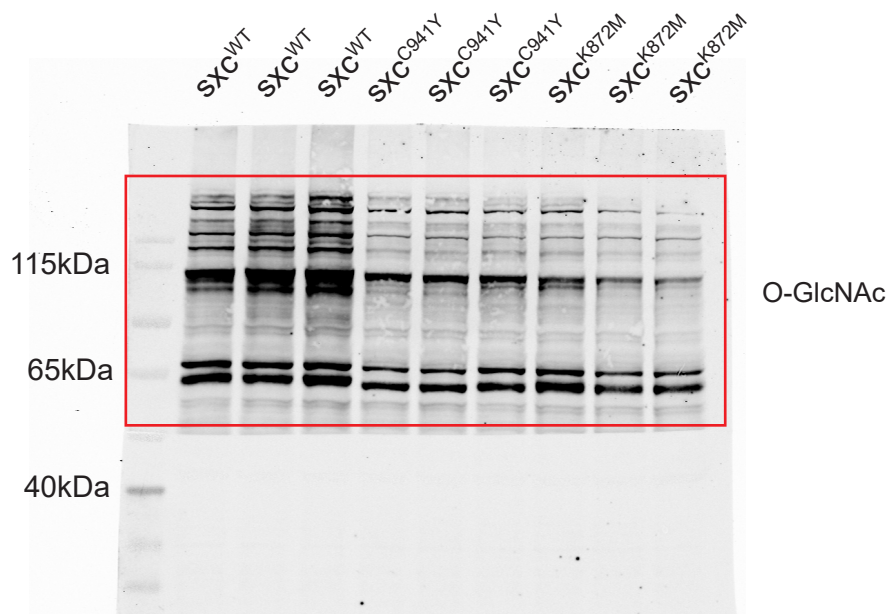

Supplement: Figure 1—source data 3. [file elife-90376-fig1-data3.zip › 1B_uncropped.pdf]

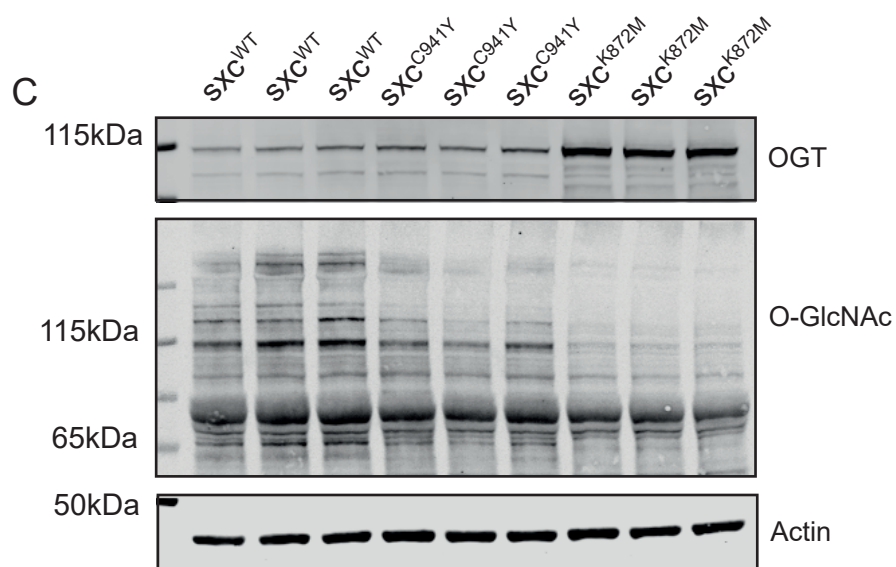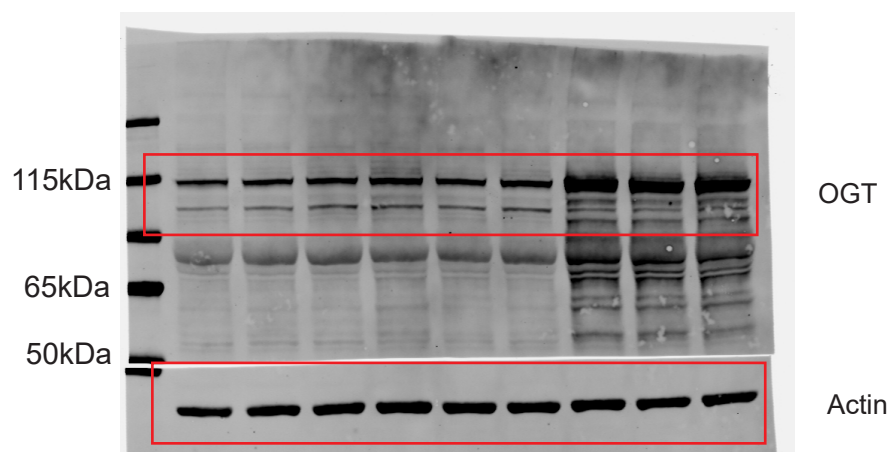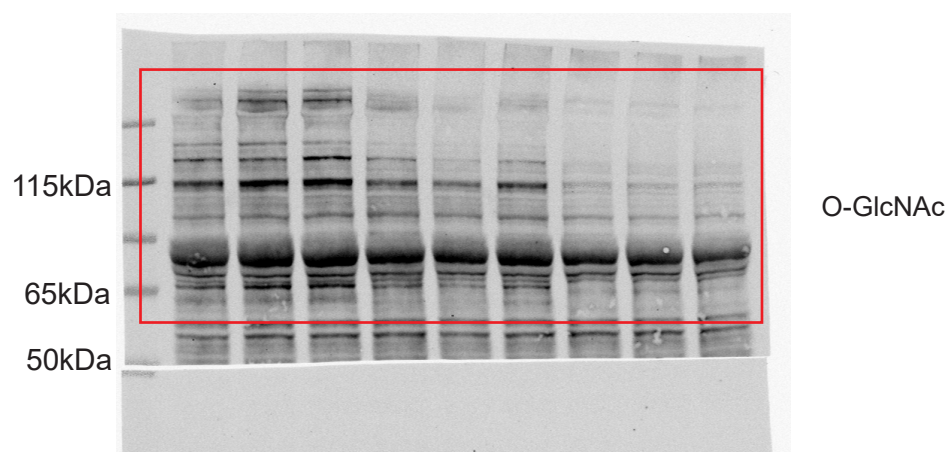

Supplement: Figure 1—source data 3. [file elife-90376-fig1-data3.zip › 1C_uncropped.pdf]

A

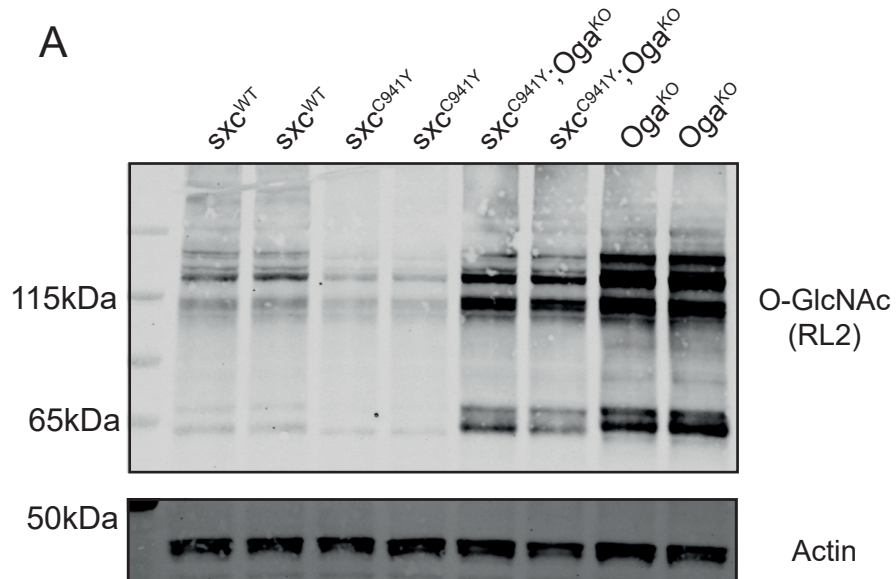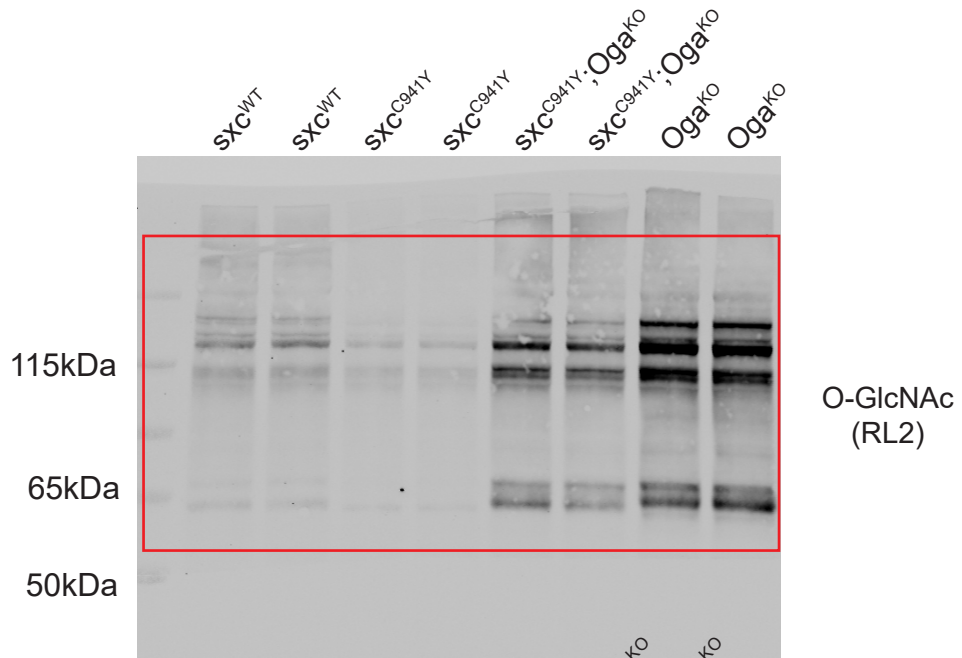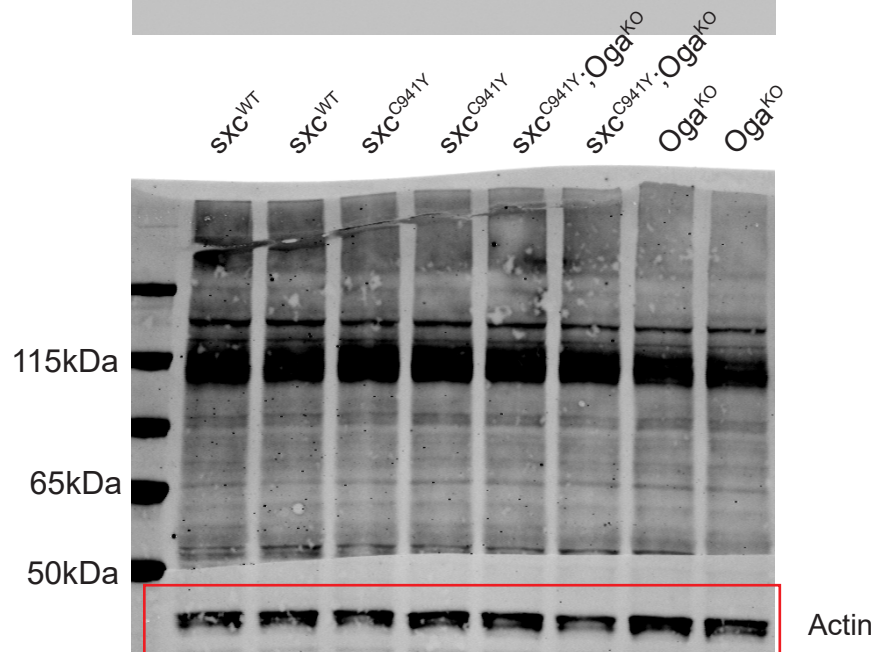

Supplement: Figure 2—source data 2. [file elife-90376-fig2-data2.zip › 2A_uncropped.pdf]

**B**

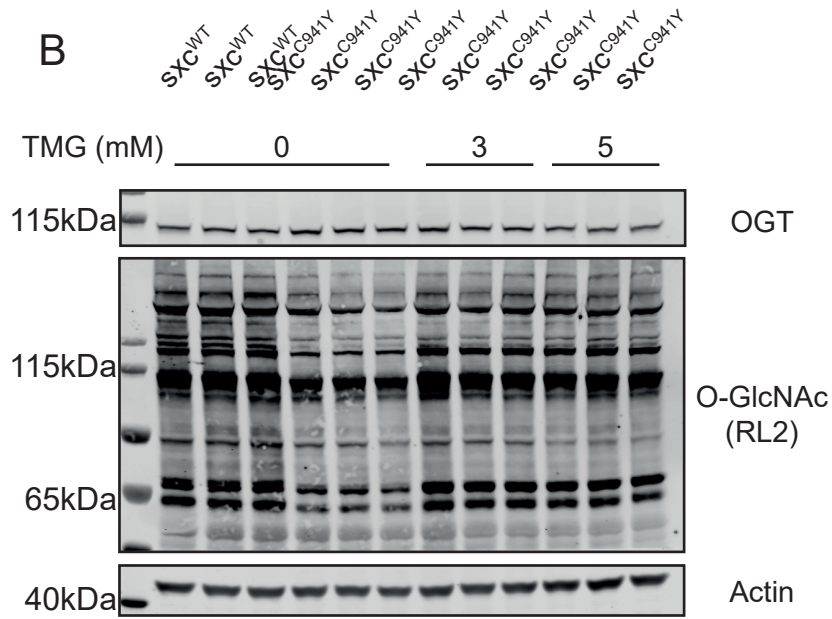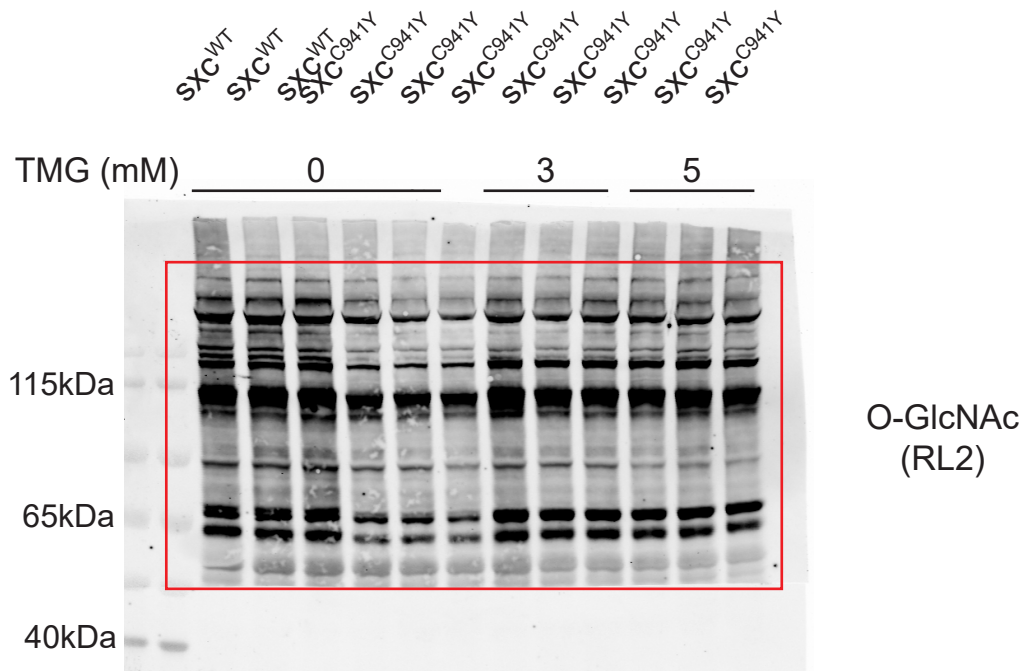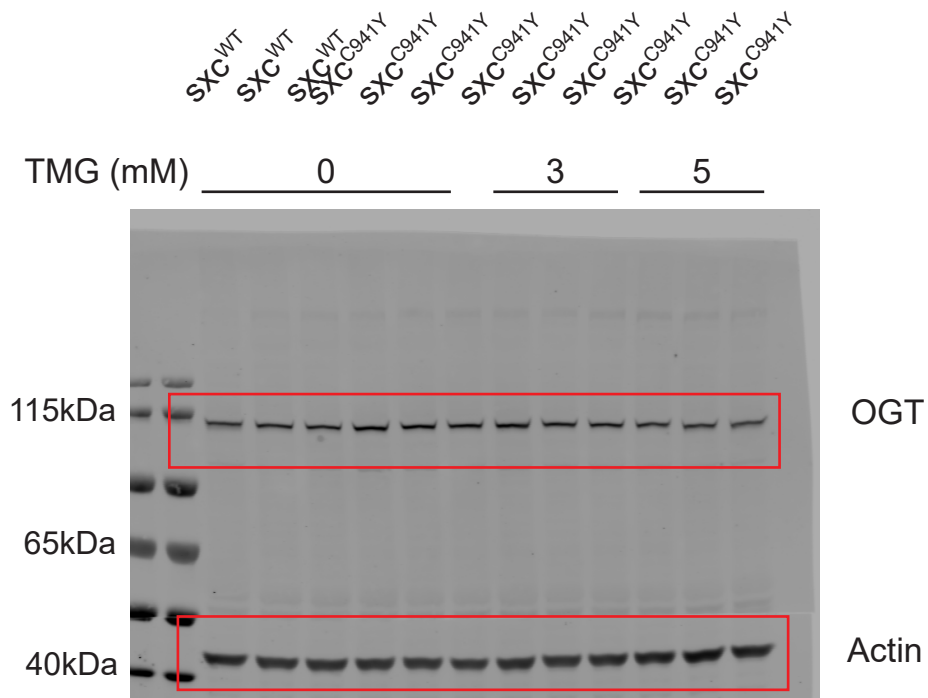

Supplement: Figure 2—source data 2. [file elife-90376-fig2-data2.zip › 2B_uncropped.pdf]

C

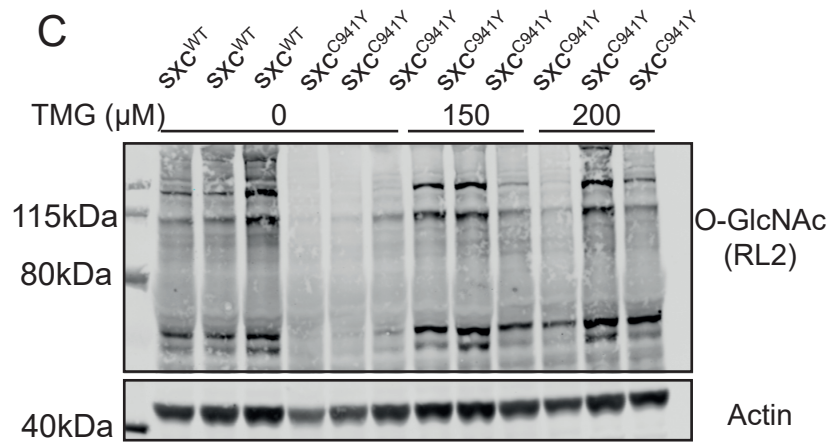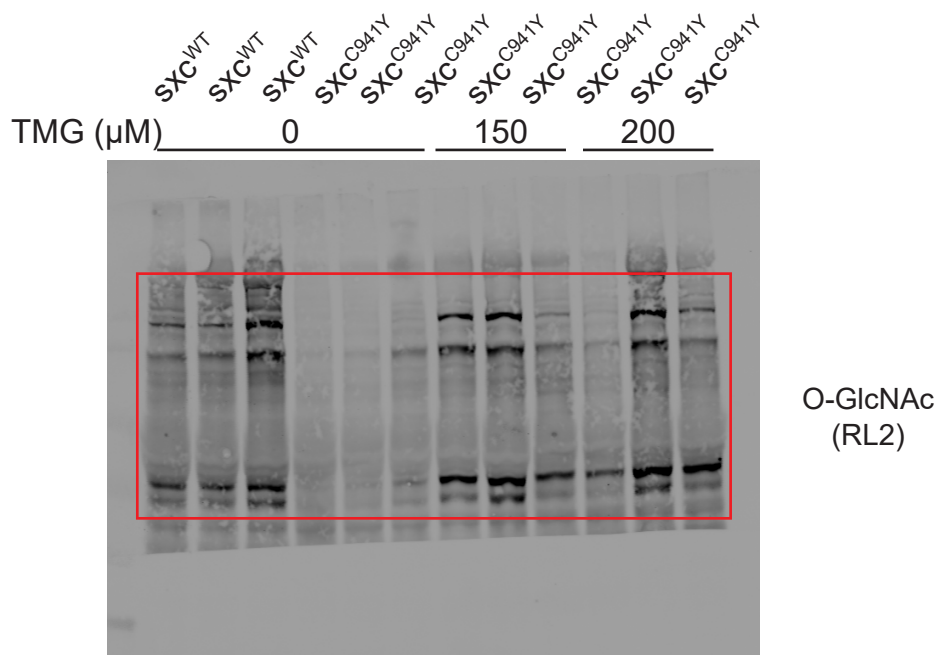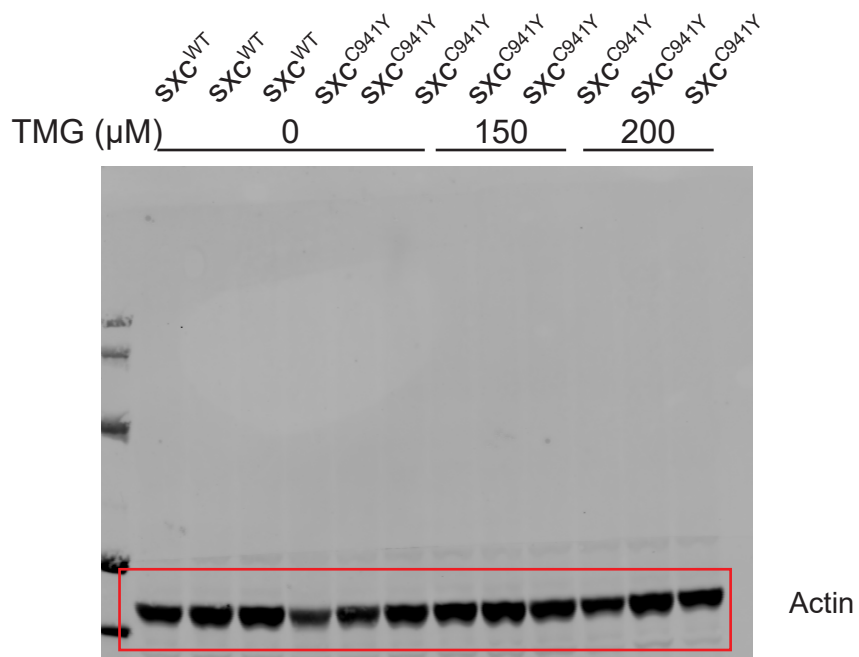

Supplement: Figure 2—source data 2. [file elife-90376-fig2-data2.zip › 2C_uncropped.pdf]
